# The effects of noninvasive brain stimulation on cognitive function in patients with mild cognitive impairment and Alzheimer's disease using resting‐state functional magnetic resonance imaging: A systematic review and meta‐analysis

**DOI:** 10.1111/cns.14314

**Published:** 2023-06-22

**Authors:** Tao Wang, Shaozhen Yan, Jie Lu

**Affiliations:** ^1^ Department of Radiology and Nuclear Medicine, Xuanwu Hospital Capital Medical University Beijing China; ^2^ Beijing Key Laboratory of Magnetic Resonance Imaging and Brain Informatics Beijing China; ^3^ Key Laboratory of Neurodegenerative Diseases Ministry of Education Beijing China

**Keywords:** Alzheimer's disease, fMRI, mild cognitive impairment, noninvasive brain stimulation

## Abstract

**Objective:**

The aim of this systematic review and meta‐analysis was to evaluate the efficacy of noninvasive brain stimulation (NIBS) on cognition using functional magnetic resonance imaging (fMRI) in patients with mild cognitive impairment (MCI) and Alzheimer's disease (AD), thus providing the neuroimaging mechanism of cognitive intervention.

**Methods:**

English articles published up to April 30, 2023 were searched in the PubMed, Web of Science, Embase, and Cochrane Library databases. We included randomized controlled trials where resting‐state fMRI was used to observe the effect of NIBS in patients with MCI or AD. RevMan software was used to analyze the continuous variables, and SDM‐PSI software was used to perform an fMRI data analysis.

**Results:**

A total of 17 studies comprising 258 patients in the treatment group and 256 in the control group were included. After NIBS, MCI patients in the treatment group showed hyperactivation in the right precuneus and decreased activity in the left cuneus and right supplementary motor area. In contrast, patients in the control group showed decreased activity in the right middle frontal gyrus and no hyperactivation. The clinical cognitive scores in MCI patients were significantly improved by NIBS, while not in AD. Some evidence regarding the modulation of NIBS in resting‐state brain activity and functional brain networks in patients with AD was found.

**Conclusions:**

NIBS could improve cognitive function in patients with MCI and AD. fMRI evaluations could be added to evaluate the contribution of specific NIBS treatment therapeutic effectiveness.

## Graphical abstract

The systematic review indicated that noninvasive brain stimulation (NIBS) intervention appeared an effective and safe treatment for patients with mild cognitive impairment and Alzheimer's disease. The improvement may be attributed to the positive modulation of NIBS on the spontaneous neural activity and cognitive network. Resting‐state functional magnetic resonance imaging evaluations could be added to target‐specific NIBS treatment to evaluate the contribution to the therapeutic effectiveness of NIBS.

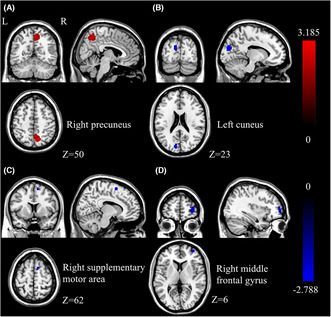

## 1 INTRODUCTION

Alzheimer's disease (AD), the most common type of dementia, is an incurable and chronic disease that is prevalent in the elderly population and can seriously affect cognitive function and basic life abilities, eventually leading to death.^1^ Patients first go through two cognitive stages, including subjective cognitive decline and mild cognitive impairment (MCI), before cognitive impairment reaches the level of dementia.^2^ Since MCI, as a prodromal stage, has a high potential to develop into AD, the consensus has been reached to focus major interventions or treatments on this population to delay the progression of cognitive impairment.^3^ However, to date, there is no systematic evidence for the effectiveness of pharmacological treatment to improve cognitive impairment and slow down the progression of dementia.^4^ The cholinesterase inhibitors, as the current most popular and preferred medical treatment, only offer short‐term and limited cognitive benefits, and their clinical value remains controversial, given the risk of side effects in clinical applications.^5^ Therefore, the effective nonpharmacological interventions are crucial for the treatment of MCI and AD, which have attracted much attention from aging researchers and neurologists.^6^

Repetitive transcranial magnetic stimulation (rTMS) and transcranial direct current stimulation (tDCS) provided two major and novel insights for noninvasive brain stimulation (NIBS) protocols, which are valuable tools for therapeutic modulation of cortical neuronal activities and showing promise as potential treatments for AD and related conditions.^7^ For rTMS, some previously published experimental studies on animal models of Parkinson's disease,^8^ Huntington's disease,^9^ and AD^10^, ^11^ as well as clinical trial models of AD^12^, ^13^ have demonstrated the beneficial therapeutic effects of TMS on neurodegenerative disorders. Some research evidence showed that rTMS can effectively improve the neuroplasticity, cortical excitability, and cognitive impairment process in MCI and AD patients.^14^ Increasing evidence indicated that more precisely worked neurochemical and neurophysiological functions would be permitted by rTMS.^15^ tDCS is another strategy of NIBS. Several tDCS studies on patients with AD, chronic insomnia, and depression have reported significant positive effects on improving clinical symptoms.^16^, ^17^, ^18^ tDCS could modulate cortical function through anodic or cathodic electrodes placed on the target scalp,^19^ which may play a crucial role in regulating neuronal function including increasing synaptic plasticity, changing cortical excitability, affecting inhibition balance, changing local cerebral blood flow, and repairing the connection between target cortex and whole‐brain network.^20^, ^21^ Increased cortical excitability and neuroplasticity might be important mechanisms for improving clinical performance and cognitive abilities in neurodegenerative diseases.^22^

Abnormalities in brain structure and function could be identified by neuroimaging technology, in particular, the changes of functional imaging, which is earlier than cognitive performance deficits to be clinically detected.^23^ With the advantages of noninvasiveness and relatively high spatial and temporal resolutions, resting‐state functional magnetic resonance imaging (rs‐fMRI) is often used to assess resting‐state brain activation in subjects, involving functional integration and separation, which is one of the recommended modalities applied to measure neuroplasticity.^24^ As an important brain imaging technique, rs‐fMRI is a valuable approach for exploring human functional neural networks.^25^ To better understand the brain properties of NIBS treatments, an increasing number of studies are applying rs‐fMRI to clinical trials. A variety of analysis methods provide neurologists and researchers with new insights for NIBS treatment of dementia, including the amplitude of low‐frequency fluctuations (ALFF), regional homogeneity (ReHo), functional connectivity (FC), and so on. However, to the best of our knowledge, the precise neuroimaging mechanism behind the effects of NIBS is not yet fully understood. No consensus has been reached, possibly due to the small sample size or distinctions of experimental results between studies. In addition, the affected brain regions observed in previous studies were diverse. Therefore, aiming to elucidate which brain area plays a crucial role in the therapeutic effect of NIBS, this systematic review and meta‐analysis integrate the results of individual neuroimaging studies to establish a clinically meaningful efficacy of NIBS for patients with MCI and AD. Understanding the mechanism of NIBS‐induced neuroplasticity can not only elucidate the core mechanism of NIBS but also provide novel ideas for the application of NIBS in the treatment of neurodegenerative diseases.

## 2 METHODS

We conducted the systematic review and meta‐analysis in line with the Cochrane Handbook for Systematic Reviews of Interventions. All procedures in this study followed the Preferred Reporting Items for Systematic Reviews and Meta‐Analyses (PRISMA) guidelines. The study was registered in the PROSPERO‐International prospective register of systematic reviews (http://www.crd.york.ac.uk/prospero/, registration number: CRD 42023387607).

### 2.1 Literature search

Studies that examined the neuroprotective effect of NIBS in MCI patients were included in the present study. A comprehensive strategy was executed on April 30, 2023 to search for pertinent literature in PubMed, Web of Science, Embase, and Cochrane Library databases. The search terms used were presented in Appendix S1. The search strategy for each database was based on its own unique characteristics. In addition, the references of included studies were manually checked to identify further studies for inclusion.

### 2.2 Inclusion and exclusion criteria

The screened articles were carefully reviewed independently by two researchers, and included in the meta‐analysis if they met the following criteria: (1) randomized controlled trials (RCTs) were conducted in patients who previously diagnosed with MCI or AD according to eligible criteria (e.g., Petersen's criteria, National Institute on Aging‐Alzheimer's Association [NIA‐AA], National Institute of Neurological and Communicative Diseases and Stroke/Alzheimer's Disease and Related Disorders Association [NINCDS‐ADRDA], Diagnostic and Statistical Manual of Mental Disorders, 5th edition [DSM‐V]); (2) rTMS or tDCS was the main intervention being investigated for the outcome differences in the treatment group while sham stimulation in the control group; (3) cognitive functions were assessed by fMRI; (4) article was published in English; (5) involved whole‐brain functional imaging in resting‐state; (6) fMRI data for quantitative analysis were displayed as three dimensional coordinates (*x*, *y*, *z*) in standard stereotactic space Talairach or Montreal Neurological Institute; (7) continuous data were displayed as mean ± standard deviation (SD), or other data types if they could be converted to mean ± SD. The exclusion criteria were as follows: (1) there was sample overlap, (2) patients who had other diseases that could cause cognitive impairment, and (3) studies without available data for analysis.

### 2.3 Quality assessment and data extraction

Two independent reviewers (T.W. and S.Z.Y.) abstracted data by using the Cochrane Collaboration's Tool for assessing the risk of bias. Any disagreement was resolved by discussion with a third reviewer (J.L.). The following criteria were used to evaluate the quality of each included trial: (1) random sequence generation, (2) allocation concealment, (3) blinding of participants and personnel, (4) blinding of outcome assessment, (5) incomplete outcome data, (6) selective reporting, and (7) other bias.

We extracted the fMRI data and neuropsychological scale scores at baseline and after the intervention of experiment groups and control groups. Data were extracted from the included studies into a standard form with respect to the name of the first author, design type, publishing year, diagnosis criteria, sample size, gender ratio, mean age, education level, mini‐mental state examination (MMSE) scores, intervention design, stimulation protocols, primary and secondary outcomes, time of follow‐up and adverse effects. Missing or unclear data for the review were obtained after corresponding with the original authors.

### 2.4 Data synthesis and meta‐analysis

A voxel‐wise meta‐analysis of regional ALFF differences between the treatment group and the control group was analyzed using the Seed‐based *d* Mapping with Permutation of Subject Images software package, version 6.21 (SDM‐PSI; http://www.sdmproject.com/). Then subgroup meta‐analyses of included studies using tDCS and rTMS was performed to investigate the possible effects of different treatment equipment. A detailed tutorial is available in the previous publications,^26^, ^27^ so we give a brief summary here. First, reported peak coordinates and effect sizes (e.g., *t* values) were extracted to prepare the peaks' text files. In the step of preprocessing, software used those files to recreate the lower and upper bounds of the possible effect‐size values of included studies. Second, a traditional random‐effects meta‐analytic method was used to derive the mean map of ALFF values, which was weighted by the sample size, variability of each study, and the between‐study heterogeneity. Both negative and positive changes are presented in the same map. We reported results using default SDM kernel size and thresholds to optimally balance false positives and negatives (uncorrected voxel *p* < 0.005, peak height *Z* > 1, cluster extent >10 voxels).

For neuropsychological scores, if the mean and SD of the score changes, after treatment relative to baseline, could not be directly extracted from the original article, they were calculated by using the following formula recommended by the Cochrane Handbook for Systematic Reviews of Interventions, Version 5.1.0 (http://www.handbook‐5‐1.cochrane.org/):

$${\text{Mean}}_{\text{change}}={\text{Mean}}_{\text{final}}-{\text{Mean}}_{\text{baseline}},$$

$${\mathrm{SD}}_{\text{change}}=\sqrt{{\mathrm{SD}}_{\text{baseline}}^{2}+{\mathrm{SD}}_{\text{final}}^{2}-\left(2\times \text{Corr}\times {\mathrm{SD}}_{\text{baseline}}\times {\mathrm{SD}}_{\text{final}}\right)}$$

### 2.5 Meta‐regression analysis

Meta‐regression analysis was conducted to examine the effects of age, gender (ratio of males and females), education, and montreal cognitive assessment (MoCA) scores. Statistical significance was determined using a stringent threshold of *p* < 0.0005 and cluster extent of >10 voxels in the meta‐regression analysis.

### 2.6 Neuropsychological scores

Mini‐mental state examination, test of strategic learning, semantic word‐generation task, auditory verbal learning test, MoCA, and repeatable battery for the assessment of neuropsychological status were extracted as outcome measures for meta‐analysis. Statistical analyses of continuous data were conducted with Reviewer Manager software, version 5.3, from the Cochrane Collaboration (RevMan; http://www.training.cochrane.org/). The effect size of NIBS on cognition was estimated by using standardized mean difference (SMD) or mean difference (MD) with 95% confidence intervals (CI). If the *I* ^2^ value was >50%, the random effect model was used for analysis. Otherwise, the fixed model was used. A forest plot was adopted to show the hypothesis test results. A statistically significant *p* value was set to 0.05.

### 2.7 Heterogeneity, publication bias and jackknife sensitivity analysis

Heterogeneity was tested by the *I* ^2^ statistic. *I* ^2^ < 50% indicates low heterogeneity. Publication bias and jackknife sensitivity analysis were not performed because the amount of included studies was less than 10.

## 3 RESULTS

### 3.1 Included studies and sample characteristics

**FIGURE 1:**
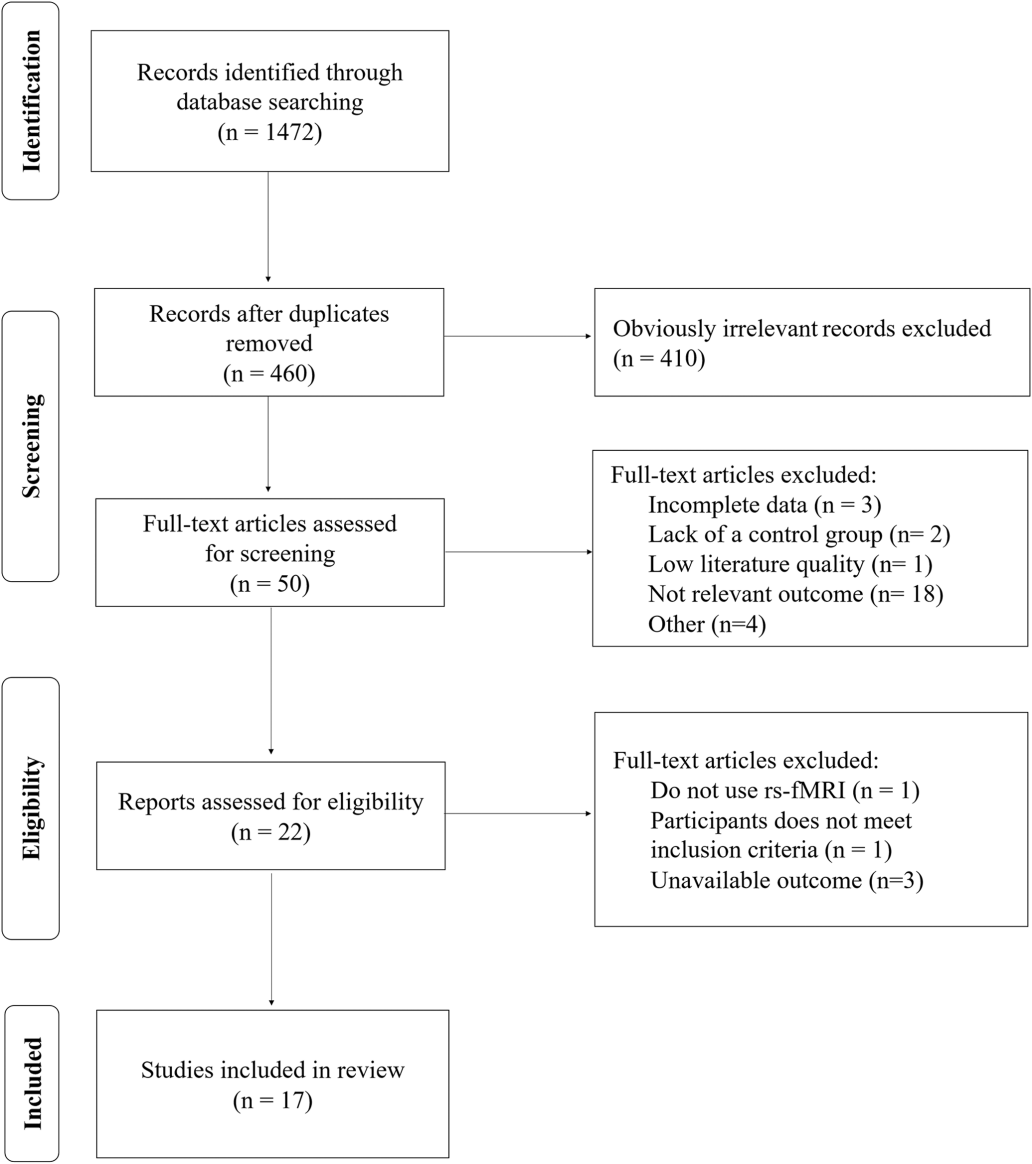
PRISMA flowchart.

By searching the four databases mentioned above, a total of 1,472 articles were found. Finally, 17 studies^28^, ^29^, ^30^, ^31^, ^32^, ^33^, ^34^, ^35^, ^36^, ^37^, ^38^, ^39^, ^40^, ^41^, ^42^, ^43^, ^44^ comprising 258 patients in the treatment group and 256 patients in the control group were identified in the review based on the inclusion and exclusion criteria, including 6 tDCS^28^, ^29^, ^30^, ^31^, ^32^, ^33^ and 11 rTMS^34^, ^35^, ^36^, ^37^, ^38^, ^39^, ^40^, ^41^, ^42^, ^43^, ^44^ papers. A flow diagram of the selection and exclusion criteria of included studies is presented in Figure 1. These studies were rated as being of good quality, shown in Figure S1. Nine studies recruited patients with MCI and 8 with AD. The stimulation target sites of NIBS included left or right, inferior frontal gyrus, dorsolateral prefrontal cortex, ventral inferior frontal gyrus, parietal cortex, precuneus, angular gyrus, and inferior parietal lobule. As for the treatment parameters of tDCS, anodal electrode plates were located on the target sites with the current density ranging from 1 to 2 mA. The duration of intervention lasted from 1 day to 4 weeks. For rTMS, 10–40 Hz stimulation coils were placed on the scalp in a tangential position with intensity varying from 40% to 100% resting motor threshold. Similarly, the treatment time lasted from 4 weeks to 3 months. Four trails reported adverse effects that were slightly transient and well tolerated. The essential characteristics of the included studies are presented in Table 1.

**TABLE 1:** Essential characteristics of the included studies.

Study	Design	Diagnosis	Diagnosis criteria	Number of participants^a^	Gender (M/F)	Mean age (Y)^a^	Education (Y)^a^	MMSE^a^
He et al.^28^	Parallel	MCI	Petersen's criteria	24	11/32	63.50 ± 4.80	10.38 ± 3.06	25.13 ± 0.79
				19		65.63 ± 3.53	9.00 ± 2.45	24.89 ± 1.10
Kang et al.^29^	Parallel	MCI	Petersen's criteria	10	13/19	77.50 ± 6.10	9.60 ± 4.40	NR
				22		72.30 ± 7.10	13.50 ± 4.90	
Meinzer et al.^30^	Cross‐over	MCI	NR	18	11/7	67.44 ± 7.27	14.33 ± 2.00	27.17 ± 1.34
				18				
Iordan et al.^31^	Cross‐over	MCI	NR	20	13/7	72.15 ± 7.14	16.65 ± 2.60	NR
				20				
Zhang et al.^32^	Parallel	MCI	DSM‐V	17	6/24	57.11 ± 3.10	NR	25.11 ± 1.27
				13		56.92 ± 2.78		25.61 ± 2.63
Pini et al.^33^	Parallel	AD	NIA‐AA	10	10/10	72.00 ± 6.00	9.00 ± 3.00	21.00 ± 2.00
				10		73.00 ± 6.00	8.00 ± 5.00	21.00 ± 2.00
Chen et al.^37^	Parallel	aMCI	NR	10	8/12	64.30 ± 7.70	11.15 ± 2.87	27.29 ± 1.59
				10				
Cui et al.^36^	Parallel	aMCI	NIA‐AA	11	8/13	73.91 ± 10.01	12.45 ± 3.98	27.73 ± 2.00
				10		74.00 ± 7.62	12.50 ± 4.07	26.50 ± 2.72
Esposito et al.^35^	Parallel	MCI	NIA‐AA	11	14/13	64.00 (60.00–74.00)^b^	13 (10.00–13.00)^b^	NR
				16		70.50 (62.50–77.25)^b^	11.00 (8.00–13.00)^b^	
Yuan et al.^34^	Parallel	aMCI	Petersen's criteria	12	11/13	65.08 ± 4.89	11.83 ± 2.37	NR
				12		64.67 ± 4.77	11.33 ± 2.15	
Velioglu et al.^38^	Parallel	AD	NR	15	5/10	69.86 ± 8.23	NR	18.09 ± 4.74
				15				
Wei et al.^39^	Parallel	AD	DSM‐V	29	16/40	70.00 ± 8.63	7.34 ± 5.54	14.48 ± 6.94
				27		71.67 ± 7.16	6.63 ± 4.99	13.74 ± 7.16
Budak et al.^40^	Parallel	AD	NINCDS‐ADRDA DSM‐V	10	5/13	72.00 ± 5.01	1, 5, 4^c^	17.50 ± 4.50
				8		74.90 ± 8.11	3, 4, 1^c^	19.62 ± 4.77
Li et al.^41^	Parallel	MCI	NINCDS‐ADRDA	10	11/17	68.40 ± 4.95	9.00 (9.00–12.75)^b^	25.50 (21.75–26.50)^b^
		AD	Petersen's criteria	18		65.78 ± 8.30	12.00 (9.00–15.00)^b^	29.00 (27.00–30.00)^b^
Liu et al.^42^	Parallel	AD	NIA‐AA	25	21/16	67.28 ± 7.74	10.44 ± 3.33	NR
				12		72.08 ± 7.30	11.66 ± 2.53	
Qin et al.^43^	Parallel	AD	NINCDS‐ADRDA	8	5/12	66.90 ± 7.40	12.30 ± 2.00	19.30 ± 6.90
				9		66.30 ± 8.10	11.50 ± 2.80	17.60 ± 5.10
Zhang et al.^44^	Parallel	AD	NIA‐AA	18	21/14	84.80 ± 5.60	NR	4.70 ± 3.90
				17		83.40 ± 4.10		3.40 ± 3.70

Study	Intervention	Sham stimulation	Stimulation site	Stimulation protocol	Outcome measures	Coordinate	Adverse effects	Follow‐up
He et al.^28^	tDCS	120 s (60 s fade‐in, 60 s fade‐out)	L. DLPFC	Anode: 1 mA, 20 min, 10 sessions, 2 weeks	ALFF, ReHo MMSE, MoCA	MNI	Mild	NR
Kang et al.^29^	tDCS	NR	L. DLPFC	Anode: 2 mA, 20 min, 10 sessions, 2 weeks	ALFF	MNI	NR	NR
Meinzer et al.^30^	tDCS	40 s (10 s fade‐in, 30 s fade‐out)	L. IFG	Anode: 1 mA, 20 min, 1 session	ECM, SWGT	NR	NO	NR
Iordan et al.^31^	tDCS	60 s (30 s fade‐in, 30 s fade‐out)	R. PC	Anode: 2 mA, 20 min, 1 session	FC	MNI	NR	NR
Zhang et al.^32^	tDCS	60 s (30 s fade‐in, 30 s fade‐out)	L. DLPFC	Anode: 1 mA, 20 min, 10 sessions, 2 weeks	TV	MNI	NR	NR
Pini et al.^33^	tDCS	Cathode	R. IPL	Anode: 1.5 mA, 25 min, 10 sessions, 2 weeks	FC	MNI	Mild	NO
Chen et al.^37^	rTMS	Angled coil	Precuneus	10 Hz, 100% RMT, 25 sessions, 4 weeks	FC GMV	MNI	NO	NR
Cui et al.^36^	rTMS	90° coil	R. DLPFC	10 Hz, 90% RMT, 10 sessions, 2 weeks	AVLT, FC	MNI	NO	2 months
Esposito et al.^35^	rTMS	Placebo coil	B. DLPFC	10 Hz, 80% RMT, 20 sessions, 4 weeks	FC, RBANS	MNI	NR	NR
Yuan et al.^34^	rTMS	90° coil	L. DLPFC	10 Hz, 80% RMT, 20 sessions, 4 weeks	ALFF, MoCA	MNI	mild	NR
Velioglu et al.^38^	rTMS	NR	L. LPFC	20 Hz, 100% RMT, 10 sessions, 2 weeks	BDNF, RSA	MNI	NR	NR
Wei et al.^39^	rTMS	45° coil	L. LPFC	10 Hz, 100% RMT, 10 sessions, 2 weeks	dFC, MMSE	MNI	Mild	3 months
Budak et al.^40^	rTMS + PT	PT	B. DLPFC	20 Hz, 10 sessions, 2 weeks	NPI, FBI, RSA	MNI	NR	1 week
Li et al.^41^	Combined intervention	rTMS ‐ alone	L. AG	20 Hz, 100% RMT, 20 sessions, 4 weeks	RSN	MNI	NR	NR
Liu et al.^42^	rTMS	Sham coil	B. AG	40 Hz, 40% RMT, 12 sessions, 4 weeks	MMSE, MoCA, FC	NR	NO	8 weeks
Qin et al.^43^	rTMS+ COG	Sham + COG	L. DLPFC	10 Hz, 100% RMT, 20 sessions, 4 weeks	ADAS‐cog ALFF, FC	MNI	NR	NR
Zhang et al.^44^	rTMS	Inclined coil	L. DLPFC	10 Hz, 100% RMT, 60 sessions, 3 months	CIBIC‐Plus, FC	MNI	NR	NR

Abbreviations: AD, Alzheimer's disease; ADAS‐cog, Alzheimer's disease assessment scale‐cognitive subscale; AG, angular gyrus; ALFF, amplitude of low‐frequency fluctuation; AVLT, auditory verbal learning test; B, bilateral; BDNF, brain‐derived neurotrophic factor; CBF, cerebral blood flow; CIBIC‐Plus, Clinician's Interview‐Based Impression of Change plus caregiver input; COG, cognitive training; dFC, dynamic functional connectivity; DLPFC, dorsolateral prefrontal cortex; DSM‐V, Diagnostic and Statistical Manual of Mental Disorders, 5th edition; ECM, eigenvector centrality mapping; F, female; FBI, Frontal Behavioral Inventory; FC, functional connectivity; GMV, gray matter volumes; IFG, inferior fontal gyrus; IPL, inferior parietal lobule; L, lefegic learning; M, male; MCI, mild cognitive impairment; MMSE, mini‐mental state examination; MNI, montreal neurological institute; MoCA, montreal cognitive assessment; NIA‐AA, National Institute on Aging‐Alzheimer's Association; NINCDS‐ADRDA, National Institute of Neurological and Communicative Diseases and Stroke/Alzheimer's Disease and Related Disorders Association; NPI, Neuropsychiatric Inventory Questionnaire; NR, not report; PC, parietal cortex; PT, pharmacological therapy; R, right; RBANS, repeatable battery for the assessment of neuropsychological status; ReHo, regional homogeneity; RSA, resting‐state activity; RSN, resting‐state networks; SWGT, semantic word‐generation task; tDCS, transcranial direct current stimulation; TOSL, test of strat; TV, temporal variability; Y, year.

^a^ Continuous data are expressed as mean ± SD. If two rows of data are displayed in a cell, the first one represents the treatment group and the other represents the control group. If only one row of data is displayed, it represents the total group.

^b^ Data are reported as median (25th, 75th percentile).

^c^ Data are reported as the number of education (not literate, 1–5 years, ≥8 years).

### 3.2 Neuropsychological scores differences in patients with MCI

Seven trials explored the effect of NIBS on neuropsychological scores in patients with MCI. Patients with MCI in the treatment group showed improved cognitive scores, compared with the control group (SMD = 1.16, 95% CI: 0.34, 1.99, *p* = 0.006). The corresponding forest plot is shown in Figure S2.

### 3.3 Regional ALFF differences in patients with MCI

**FIGURE 2:**
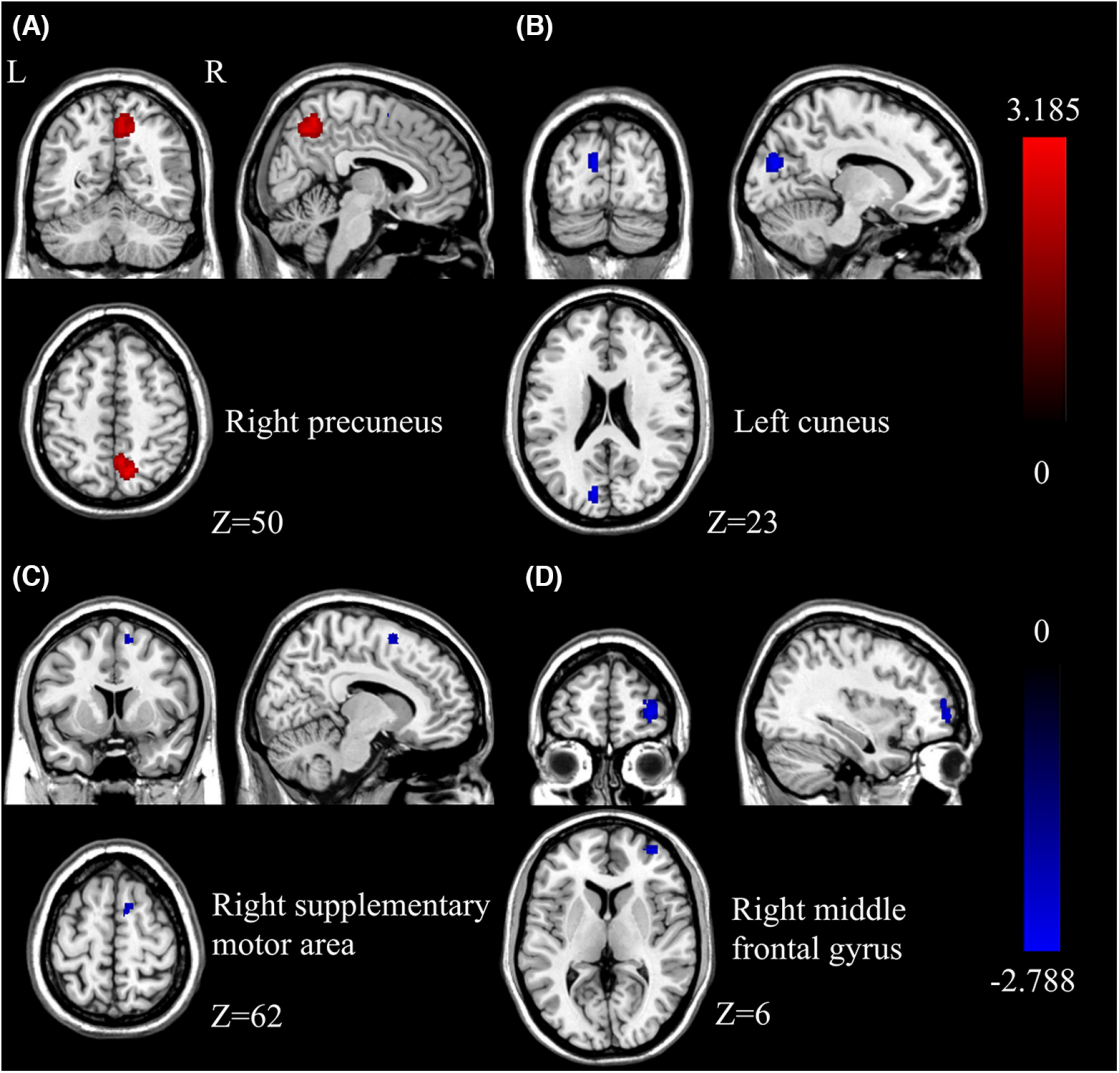
SDM‐PSI meta‐analysis for the amplitude of low‐frequency fluctuation change in the experimental group (A–C) and control group (D) patients with MCI after NIBS treatment. Red color refers to hyperactivation, and blue color refers to hypoactivation. L, left; R, right.

Three studies were included in the meta‐analysis. Compared to baseline, patients in the treatment group showed hyperactivation in the right precuneus (*p* < 0.005, *Z* = 3.185) after NIBS therapy and the decreased activity of the left cuneus (*p* < 0.005, *Z* = −2.723) and right supplementary motor area (*p* < 0.005, *Z* = −2.635). Patients in the control group showed no hyperactivation and a decrease of activity in the right middle frontal gyrus (*p* < 0.005, *Z* = −2.788). Peak coordinates and cluster breakdowns are shown in Table 2. Differences in the brain regions between the treatment group and the control group have been visualized in Figure 2.

**TABLE 2:** ALFF changes in patients with MCI after NIBS treatment compared to baseline.

	Anatomical label	MNI coordinates			SDM‐*Z* score	*p* value	Voxels	*I* ^2^ (%)
		*X*	*Y*	*Z*				
Treatment group	R Precuneus	10	−64	50	3.185	<0.005	464	6.95
	L Cuneus	−12	−82	20	−2.723	<0.005	161	3.69
	R SMA	12	8	62	−2.635	<0.005	44	1.95
Control group	R MFG	34	56	2	−2.788	<0.005	131	0.54

Abbreviations: ALFF, amplitude of low‐frequency fluctuations; L, left; MCI, mild cognitive impairment; MFG, middle frontal gyrus; MNI, montreal neurological institute; R, right; SDM, Seed‐based *d* Mapping; SMA, supplementary motor area.

### 3.4 Subgroup analysis in patients with MCI

Patients after tDCS showed hyperactivation in the right precuneus (*p* < 0.005, *Z* = 3.177) and decreased activity of the left cuneus (*p* < 0.005, *Z* = − 3.504). Patients after rTMS showed hyperactivation in the right middle frontal (*p* < 0.005, *Z* = 3.970). The corresponding peak coordinates and cluster breakdowns are shown in Table S1.

### 3.5 Meta‐regression analysis in patients with MCI

Meta‐regression analysis revealed that increasing age (*p* < 0.0005, *Z* = 3.888) and MoCA scores (*p* < 0.0005, *Z* = 3.305) was associated with hyperactivation in the right supramarginal gyrus (Table S2).

### 3.6 Heterogeneity analysis in patients with MCI

In the treatment group, the right precuneus, left cuneus, and right supplementary motor area showed low between‐study heterogeneity of effect size differences in peak coordinates (*I* ^2^ = 1.95%–6.95%) and low heterogeneity in the control group (*I* ^2^ = 0.54%). The heterogeneity results are shown in Table 2.

### 3.7 FC in patients with MCI

**FIGURE 3:**
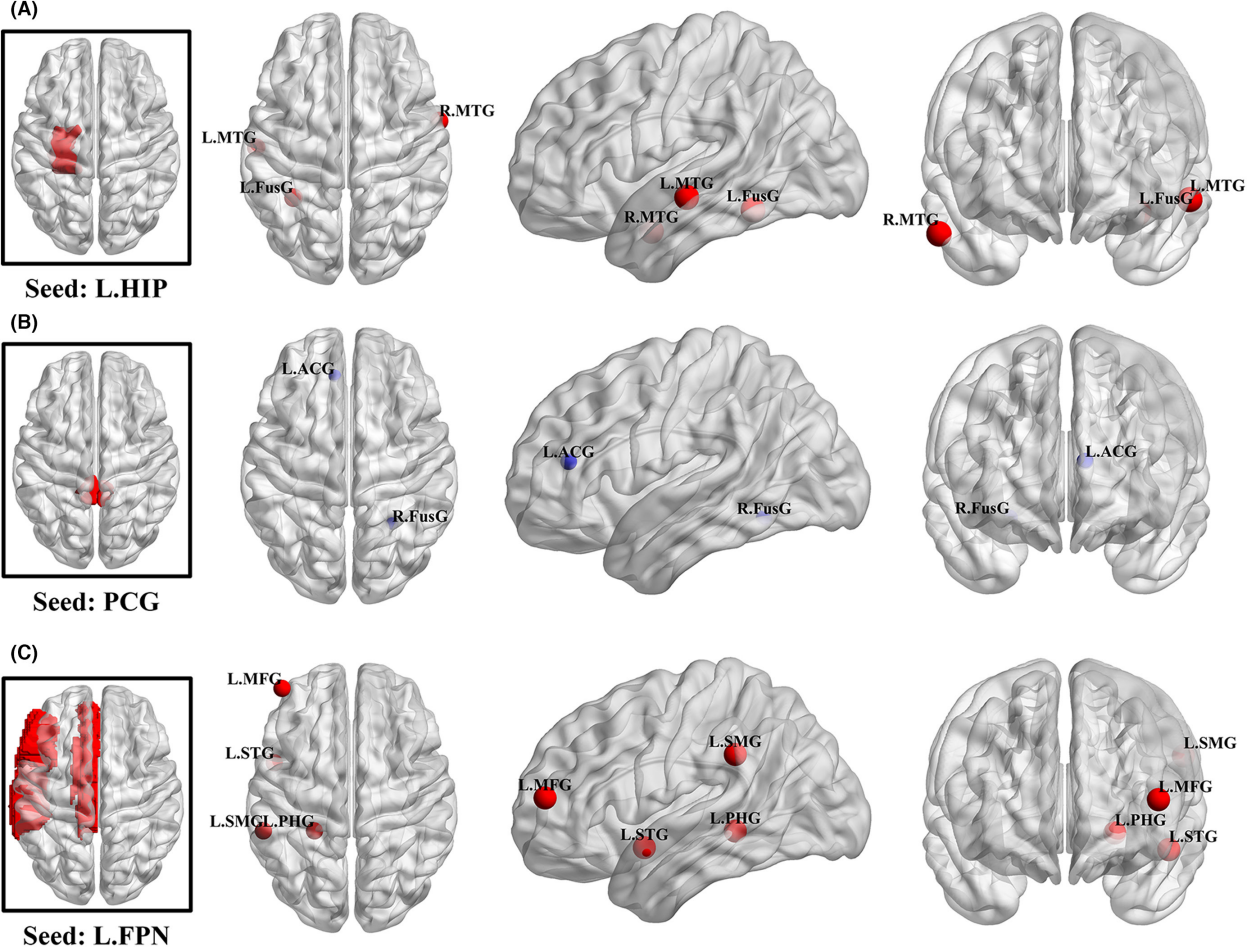
The change of resting‐state functional connectivity after NIBS intervention in the review. (A) Chen et al.,^37^ (B) Cui et al.,^36^ (C) Esposito et al.^35^ Node with red color refers to increase, and blue color refers to decrease. ACG, anterior cingulate gyrus; FPN, frontoparietal networks; FusG, fusiform gyrus; HIP, hippocampus; L, left; MFG, middle frontal gyrus; MTG, middle temporal gyrus; PCG, posterior cingulate gyrus; PHG, parahippocampal gyrus; R, right; SMG, supramarginal gyrus; STG, superior temporal gyrus.

The differences of FC in MCI patients were investigated directly in five trails. Given the differences in metrics, quantitative meta‐analysis seems impossible. The results of included articles were summarized as follows. rTMS was applied in three studies. Based on the collective descriptions in the literature of rTMS, the seed‐based region of interest (ROI) were placed on left frontoparietal networks (L.FPN),^35^ posterior cingulate gyrus (PCG),^36^ and left hippocampus,^37^ respectively. Specifically, the first one showed increased FC in the left supramarginal gyrus, middle frontal gyrus, superior temporal gyrus, and parahippocampal gyrus. Another study that investigated the connectivity with PCG reported lower FC in the left anterior cingulate gyrus and right fusiform gyrus after intervention. As for connectivity with left hippocampus, it showed a significantly increase in bilateral middle temporal gyrus and left fusiform gyrus. A summary of the changed FC induced by rTMS in these areas is depicted in Figure 3. In addition, two RCTs investigated the beneficial effects of tDCS on networks in patients with MCI using some novel analytical methods of fMRI. One reported that increased network segregation in MCI patients was driven by the effects of tDCS on the associated brain networks, specifically the default mode network (DMN) and dorsal attention network.^31^ The other reported that the temporal variability of dynamic functional connectivity (dFC) in regions belonging to DMN, central executive network, and salience network was significantly altered after tDCS.^32^

### 3.8 MMSE in patients with AD

Three studies employing the MMSE were included in the meta‐analysis. No significantly improved MMSE score in patients with AD in the treatment group after NIBS compared with the control group (MD = 0.88, 95% CI: −1.65, 3.42, *p* = 0.49). The corresponding forest plot is shown in Figure S3.

### 3.9 Resting‐state activity differences in patients with AD

Three included trials investigated the effect of NIBS on modulating the resting‐state activity differences in patients with AD. It also seems impossible to conduct a quantitative meta‐analysis in consideration of the different metrics. One study reported decreased resting‐state activity in the precuneus and increased in the right frontal opercular cortex after the intervention compared with before the rTMS intervention, determining fusiform gyrus and lateral occipital cortex as ROIs.^38^ Another RCT indicated that after rTMS intervention, resting‐state activation significantly increased in DMN, comprised of the middle temporal gyrus, central opercular cortex, intracalcarine, superior parietal lobule, and paracingulate cortex.^40^ Similarly increased activation had also been reported previously, while decreased in the left middle frontal gyrus.^43^

### 3.10 FC in patients with AD

Four RCTs reported the changes of FC in patients with AD after NIBS. Wei et al.^39^ reported a significantly higher dFC magnitude of DMN in patients in the treatment group, compared with the sham group, after rTMS intervention. Moreover, rTMS significantly increased intranetwork connectivity of FPN^41^ and enhanced local and global functional integration within bilateral angular gyrus.^42^ As well as, one RCT reported that the patients after intervention showed significant FC differences in DMN between anodal and cathodal stimulation, although the differences before and after treatment were not statistically significant.^33^

## 4 DISCUSSION

It is suggested that NIBS therapy could alleviate neurological deficits and improve cognitive performance in patients with MCI and AD. The improvements in neuropsychological scores have been discussed in some previous meta‐analysis studies,^13^, ^45^, ^46^ so we will not discuss them further here. In this paper, we mainly focus on the changes in fMRI after NIBS intervention. In the real treatment group, MCI patients showed hyperactivation in the right precuneus and hypoactivation in the left cuneus and right supplementary motor area. In the sham treatment group, patients showed decreasing brain activities in the right middle frontal gyrus. Patients after tDCS showed hyperactivation in the right precuneus and hypoactivation in the left cuneus, and after rTMS showed hyperactivation in the right middle frontal. In the part of the literature review of rs‐fMRI, the DMN is the best characterized and most concerned among the functionally coordinated networks in patients with MCI and AD, while FPN is also worthy of researchers' attention.

Most of the basal activity appears to be mediated by neurons that are continuously active and participate in DMN, also known as the resting‐state network or the negative task network.^47^ Although the exact relationship between structural and FC is not always straightforward, DMN is anatomically defined by special connections between multiple centers, including the PCG, adjacent precuneus, medial prefrontal cortex, and part of the parietal cortex.^48^, ^49^ DMN dysfunction has been well established in AD and documented in both preclinical stages and at‐risk subjects, thus representing a potential disease target of clinical intervention and observation.^50^ Increasing clinical evidence suggests that local brain activity and FC within DMN, especially the posterior part, are disrupted in association with memory impairment in MCI and AD patients.^51^ The changes in the brain are associated with the severity and progression of AD.^52^, ^53^ In the preclinical stages of AD, hippocampal formations are dissociated from DMN function, as DMN function and its interactions are disrupted in several important regions involving hippocampal formations, especially between the PCG and hippocampal formations.^54^, ^55^ A previous meta‐analysis showed aberrant spontaneous low‐frequency brain activity in the posterior DMN of MCI patients, including hypoactivation in the bilateral precuneus/PCG, the right supramarginal gyrus, and so on, compared with healthy elders.^56^ In our review, the obvious hyperactivation in the precuneus after intervention indicated that NIBS could improve cognitive function via altering neuronal activities in DMN. Included studies on FC further illustrated this effect. Some studies have demonstrated the efficacy of NIBS in modulating DMN connectivity and improving episodic memory in early AD.^32^, ^57^, ^58^ DMN is the first brain network associated with amyloid deposition in early AD, and the protein deposition affects FC between these regions and others.^59^ Although DMN is deeply involved in the aging process and the pathological evolution of cognitive function, changes over time reflect alterations in interconnectivity (increase or decrease) revealed by different stages of FC.^60^, ^61^, ^62^, ^63^ An FC reduction in DMN is generally considered a biomarker of functional decline in AD, but interpretations of the changes in bidirectional connectivity observed in different studies of MCI patients, especially enhanced interactions with PCG, have been variously explained.^36^ The increased FC could be explained as a “compensatory process” of the brain aiming to make up for brain regions with hypoactivation. Therefore, it is a reasonable argument that increased or decreased FC could be observed after NIBS treatment. It still needs more clinical trials to prove. In addition, alteration in precuneus/PCG connectivity showed the most consistency across the study, yet it was not present in all related studies. FPN, including DLPFC and posterior parietal cortex, is another crucial cognition‐related network and plays an important role in top‐down control of attention and cognitive modulation in patients with MCI and AD.^41^ The observed immediate and long‐term improvements of NIBS treatment suggested that potential effects on the FC of FPN in some regions are crucial for executive function. Furthermore, a more extensive left FPN was associated specifically with verbal fluency function.^64^ Improvements in executive and language function in patients with MCI and AD after NIBS may be supported by changes in the FC of FPN. Further investigation of these important brain regions is warranted.

Hippocampus, another vulnerable brain region in patients with MCI and AD, is an important brain structure connecting the anterior temporal and posterior medial regions of the medial temporal system.^65^ A significant decrease in resting‐state FC between the hippocampus and medial prefrontal cortex is an important feature of AD patients. The hippocampus is an important driving force affecting brain functional activity, while medial prefrontal cortex is an important integration force for converging information.^66^ Pathway between the two plays a crucial role in memory, learning abilities, and other advanced cognitive functions.^67^ Pathological studies of AD have shown that neurofibrillary tangles were first found in medial temporal lobe structures, and the hippocampus was one of the first brain regions to be affected.^68^ A previous study combined two well‐established techniques (fMRI and rTMS) to investigate the causal relationship between neuroimaging functional changes of the hippocampus and episodic memory impairment in patients with MCI. And the results showed that rTMS could ameliorate episodic memory deficits of MCI by directly modulating the precuneus–hippocampus–middle temporal gyrus circuit.^37^ In addition, the functional changes of the hippocampus could be used as an important target for the dynamic cognitive follow‐up of AD patients.

Clinical efficacy and cognitive prognosis could be directly affected by differences in the stimulation protocols of NIBS. High frequency (not less than 10 Hz) rTMS, below resting motor threshold, more than 10 sessions, and anodal tDCS, 1–2 mA, one or more than 10 sessions, were applied in the included studies. Although dorsolateral prefrontal cortex (DLPFC) is the most popular stimulation site in the brain, other brain regions are worthy to be explored, such as inferior fontal gyrus, angular gyrus. Some previous studies confirmed that high‐frequency rTMS over the left DLPFC and low‐frequency rTMS over the right DLPFC could improve memory functions, and high‐frequency rTMS targeting the right inferior frontal gyrus enhanced executive performance in patients with MCI and AD.^13^, ^69^ For tDCS, anodal stimulation with number was greater than 10 and the current density was 2.5 mA/cm^2^ seems to be more efficacious.^19^ However, all current studies on optimal stimulation parameters of NIBS were based on clinical scores rather than objective imaging evidence. Therefore, it is worth to conduct further experiments based on neuroimaging to determine the optimal stimulation parameters and maximize clinical benefit in MCI and AD patients.

Several limitations of the study should be noted. First, data could not be pooled since the high heterogeneity in terms of the stimulation parameters among the studies (e.g., target cerebral area, including inferior frontal gyrus, DLPFC, precuneus, angular gyrus, and so on). Thus, the optimized stimulation protocols in the treatment of MCI or AD remain to be tested in future studies. Second, a relatively small sample size was included in the analysis. Third, the SDM is based on the coordinates from published studies rather than on raw data, which limits its accuracy. Finally, the current analysis could not determine whether MCI would progress to AD or not. Future longitudinal studies should investigate the effect size after NIBS intervention.

## 5 CONCLUSIONS

The current systematic review found that NIBS intervention appeared an effective and safe treatment for patients with MCI and AD. The improvement may be attributed to the positive modulation of NIBS on the spontaneous neural activity and cognitive network. fMRI evaluations could be added to target‐specific NIBS treatment to evaluate the contribution to the therapeutic effectiveness of NIBS. These findings provide new insights into the neuroimaging mechanisms underlying efficacy of NIBS.

## AUTHOR CONTRIBUTIONS

We acknowledge the contributions of each author. The author contributions were displayed as follows: Study design: Tao Wang and Shaozhen Yan. Data collection, analysis, and interpretation: Tao Wang, Shaozhen Yan, and Jie Lu. Manuscript drafting: Tao Wang. Critical revision of the manuscript: Shaozhen Yan and Jie Lu.

## CONFLICT OF INTEREST STATEMENT

The authors declare no potential conflict of interest.

## Supporting information


Appendix S1

## Data Availability

Data sharing is not applicable to this article as no new data were created in this study.
