# Supplementary material for: The effects of noninvasive brain stimulation on cognitive function in patients with mild cognitive impairment and Alzheimer's disease using resting‐state functional magnetic resonance imaging: A systematic review and meta‐analysis
Source: CNS Neurosci Ther. 2023 Jun 22;29(11):3160–72. doi: 10.1111/cns.14314 (PMC10580344; doi:10.1111/cns.14314)
Supplement: Supplementary file 1 — Appendix S1 [file CNS-29-3160-s001.zip › Supplementary_Material_for_Review (9)-2.docx]

**Supplementary Material for Review**

The search terms were #1 AND #2 AND #3.

#1: “mild cognitive impairment” OR “MCI” OR “cognitive deficit” OR “cognitive impairment” OR “Alzheimer's disease” OR “AD”

#2: “repetitive transcranial magnetic stimulation” OR “rTMS” OR “transcranial magnetic stimulation” OR “TMS” OR “transcranial direct current stimulation” OR “tDCS” OR “transcranial electrical stimulation” OR “tES” OR “noninvasive brain stimulation” OR “NIBS”

#3: “neuroimaging” OR “functional magnetic resonance imaging” OR “fMRI” OR “resting state” OR “default network” OR “brain network” OR “amplitude of low-frequency fluctuation” OR “ALFF” OR “regional homogeneity” OR “ReHo” OR “functional connectivity” OR “FC”.

**
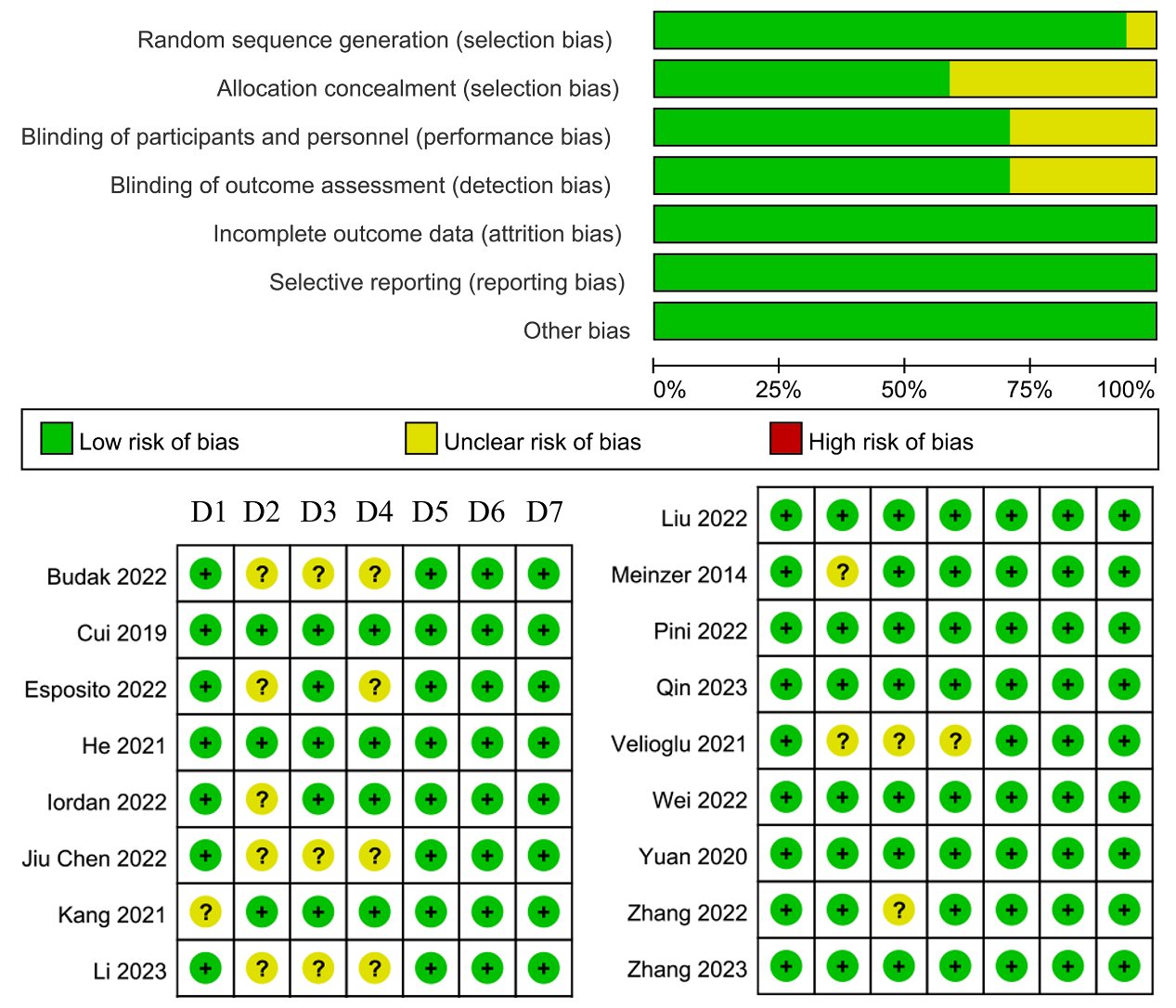
**

**FIGURE S1** Risk of bias summary. D1, random sequence generation; D2, allocation concealment; D3, blinding of participants and personnel; D4, blinding of outcome assessment; D5, incomplete outcome data; D6, selective reporting; D7, other bias.


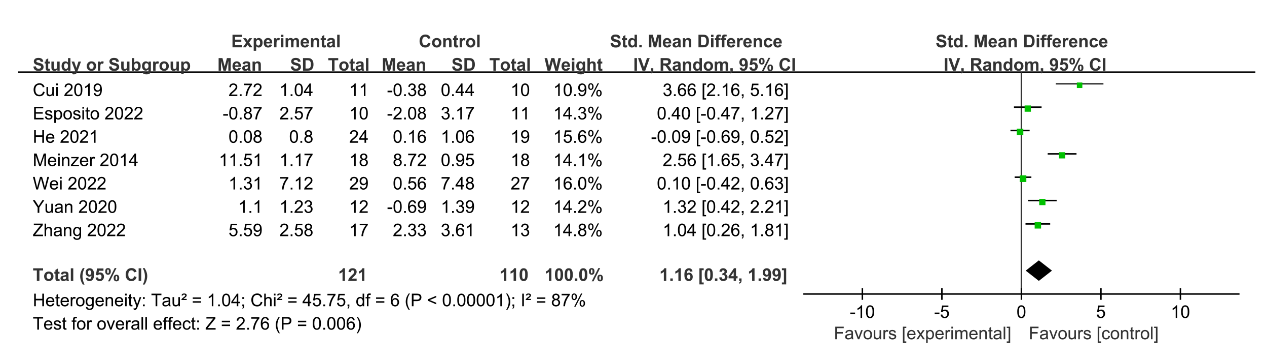


**FIGURE S2** Forest plot for the pooled effect of NIBS on neuropsychological scores after intervention. SD, standard deviation; CI, confidence interval.

**
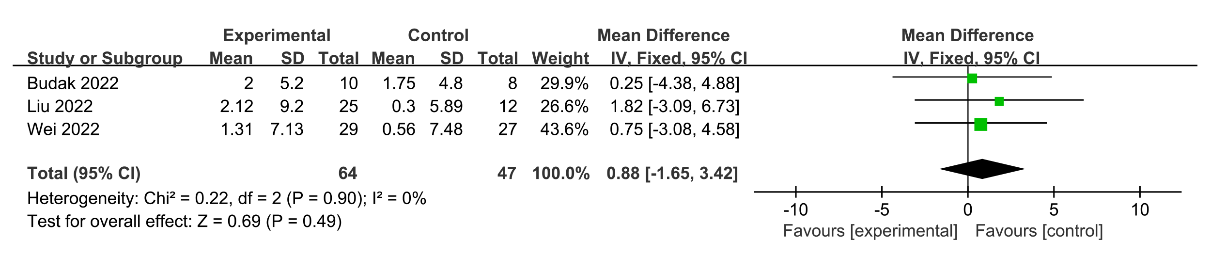
**

**FIGURE S3** Forest plot for the pooled effect of NIBS on MMSE after intervention. SD, standard deviation; CI, confidence interval.

**TABLE S1** Results for subgroup analysis

|  | Anatomical label | MNI  coordinates | | | SDM-Z score | *P* value | Voxels | *I^2^* (%) |
| --- | --- | --- | --- | --- | --- | --- | --- | --- |
|  |  | X | Y | Z |  |  |  |  |
| tDCS |  |  |  |  |  |  |  |  |
|  | R Precuneus | 4 | -56 | 54 | 3.177 | <0.005 | 218 | 0.01 |
|  | L Cuneus | -10 | -82 | 22 | -3.504 | <0.005 | 208 | 0.00 |
| rTMS |  |  |  |  |  |  |  |  |
|  | R MFG | 45 | 30 | 21 | 3.970 | <0.005 | 102 | 0.00 |

Abbreviations: MNI, montreal neurological institute; R, right; L, left; MFG, middle frontal gyrus; SDM, Seed-based *d* Mapping.

**TABLE S2** Meta-regression analysis in treatment group

|  | Anatomical label | MNI  coordinates | | | SDM-Z score | *P* value | Voxels |
| --- | --- | --- | --- | --- | --- | --- | --- |
|  |  | X | Y | Z |  |  |  |
| Effect of age |  |  |  |  |  |  |  |
|  | R SMG | 60 | -36 | 32 | 3.888 | <0.0005 | 248 |
| Effect of MoCA |  |  |  |  |  |  |  |
|  | R SMG | 62 | -42 | 34 | 3.305 | <0.0005 | 46 |

Abbreviations: MoCA, montreal cognitive assessment; MNI, montreal neurological institute; R, right; SMG, supramarginal gyrus; SDM, Seed-based *d* Mapping.
