# Supplementary figures and images for: The effects of noninvasive brain stimulation on cognitive function in patients with mild cognitive impairment and Alzheimer's disease using resting‐state functional magnetic resonance imaging: A systematic review and meta‐analysis
Source: CNS Neurosci Ther. 2023 Jun 22;29(11):3160–72. doi: 10.1111/cns.14314 (PMC10580344; doi:10.1111/cns.14314)

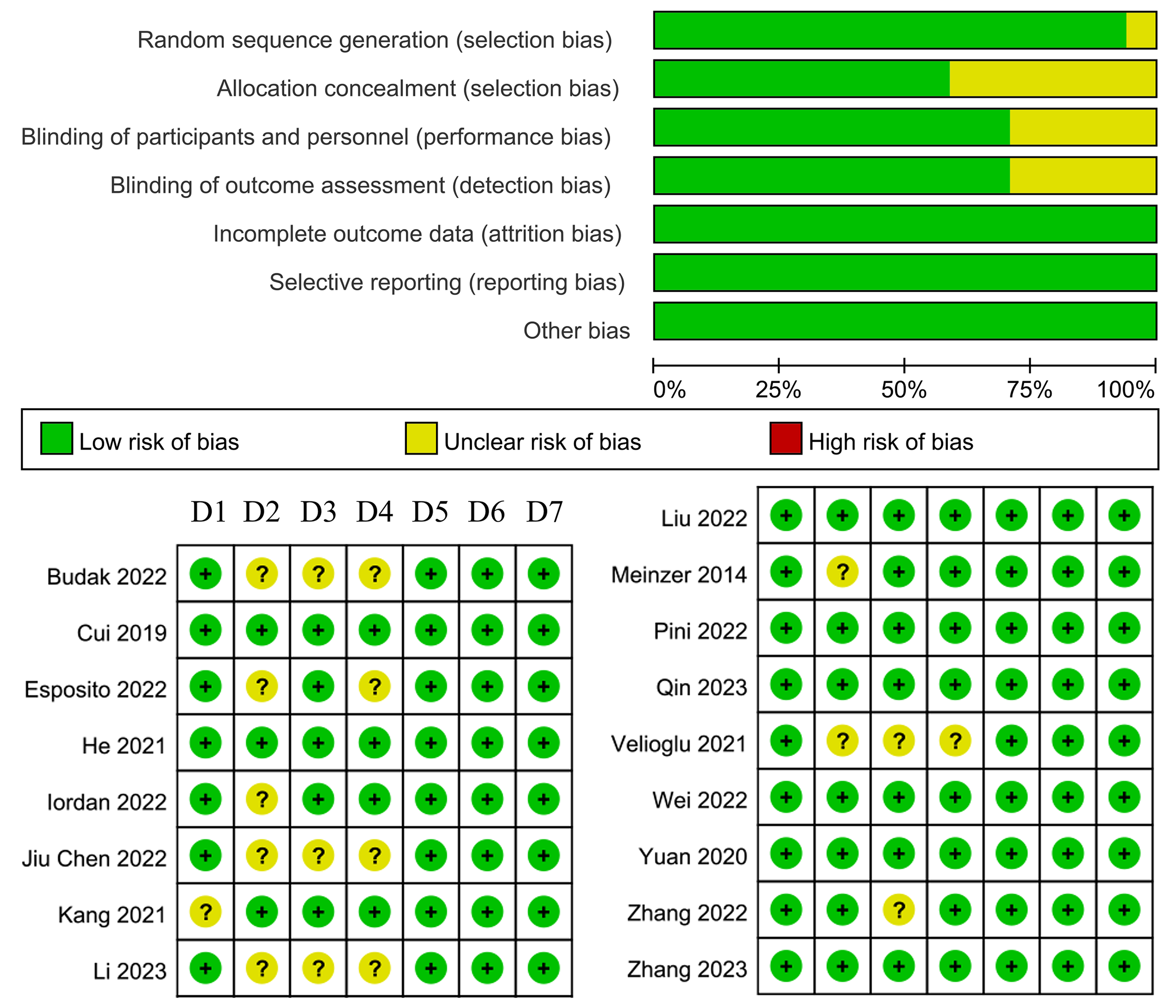

Supplement: Supplementary file 1 — Appendix S1 [file CNS-29-3160-s001.zip › Figure S1.tif]

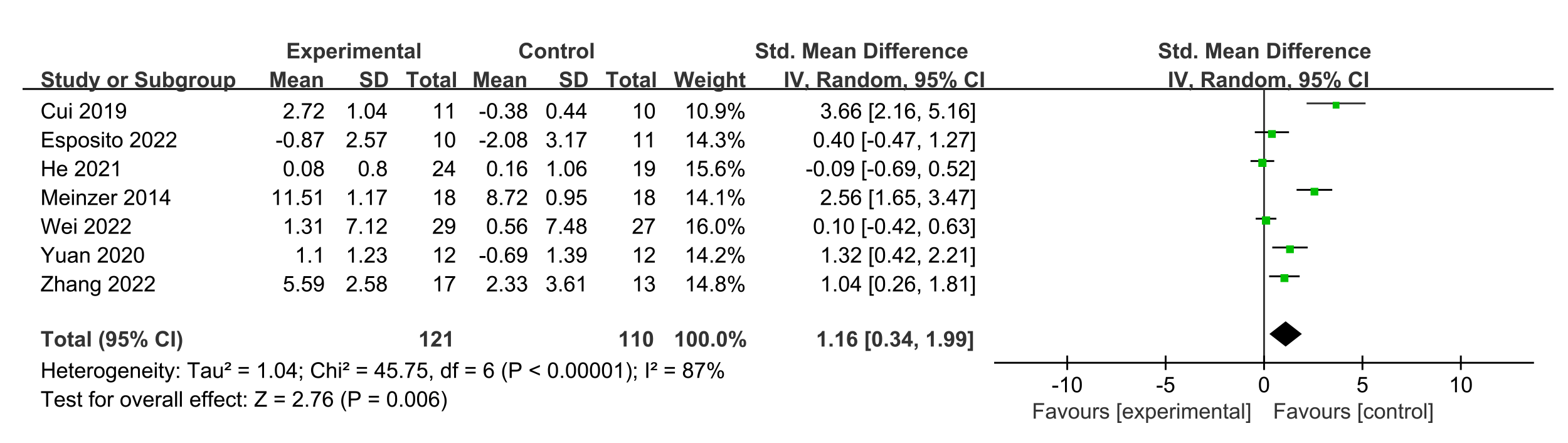

Supplement: Supplementary file 1 — Appendix S1 [file CNS-29-3160-s001.zip › Figure S2.tif]

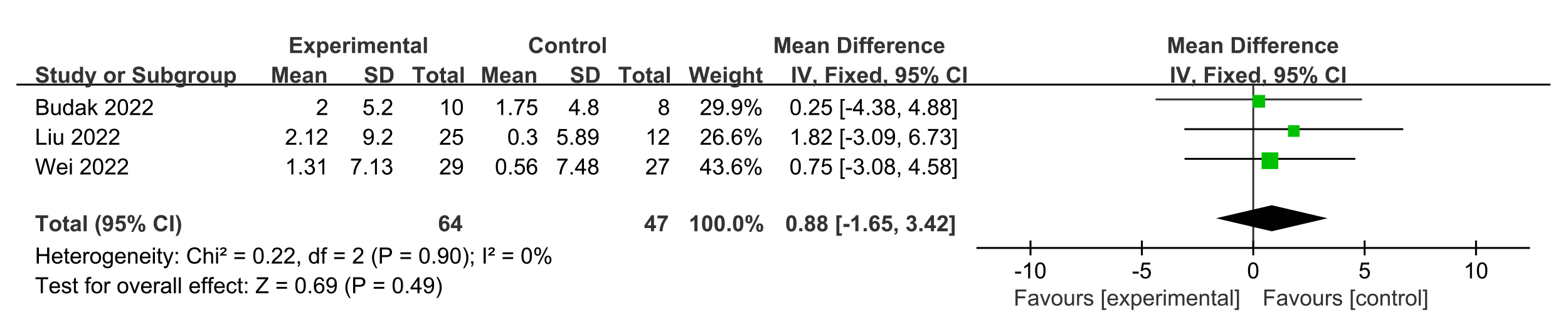

Supplement: Supplementary file 1 — Appendix S1 [file CNS-29-3160-s001.zip › Figure S3.tif]
